# Engineering Microbes for Plant Polyketide Biosynthesis

**DOI:** 10.5936/csbj.201210020

**Published:** 2013-02-22

**Authors:** François-Xavier Lussier, David Colatriano, Zach Wiltshire, Jonathan E. Page, Vincent J. J. Martin

**Affiliations:** a Department of Biology, Centre for Structural and Functional Genomics, Concordia University, 7141 Sherbrooke Street West, Montréal, Québec, Canada, H4B 1R6; b National Research Council of Canada, 110 Gymnasium Place, Saskatoon, Saskatchewan, Canada, S7N 0W9

**Keywords:** Plant polyketide, metabolic engineering, microbes

## Abstract

Polyketides are an important group of secondary metabolites, many of which have important industrial applications in the food and pharmaceutical industries. Polyketides are synthesized from one of three classes of enzymes differentiated by their biochemical features and product structure: type I, type II or type III polyketide synthases (PKSs). Plant type III PKS enzymes, which will be the main focus of this review, are relatively small homodimeric proteins that catalyze iterative decarboxylative condensations of malonyl units with a CoA-linked starter molecule. This review will describe the plant type III polyketide synthetic pathway, including the synthesis of chalcones, stilbenes and curcuminoids, as well as recent work on the synthesis of these polyketides in heterologous organisms. The limitations and bottlenecks of heterologous expression as well as attempts at creating diversity through the synthesis of novel “unnatural” polyketides using type III PKSs will also be discussed. Although synthetic production of plant polyketides is still in its infancy, their potential as useful bioactive compounds makes them an extremely interesting area of study.

## Introduction

Through the process of natural selection, plants have evolved the ability to produce an array of defensive chemicals. Plant secondary metabolism, also termed specialized metabolism, represents a unique and vast pool of highly diversified molecules that qualitatively and quantitatively vary greatly within and between species [1]. While secondary metabolites are not directly involved in essential cellular functions, they play a major role in the adaptation of plants to their environment [2]. The impressive synthetic capacity of plants has long been exploited by mankind as a source of colorants, flavours, fragrances, traditional medicines and pharmaceutical drugs [3]. Presently, plant secondary metabolites represent valuable compounds for the pharmaceutical, cosmetic, agro-food, and fine chemicals industries [4].

Among plant secondary metabolites, polyketides represent a large group of structurally diverse molecules. In plants, polyketides are synthesized by type III polyketide synthases (PKSs) by the condensation of acetyl (ketide) units with a coenzyme A (CoA)-linked starter molecule [5]. The structural diversity of the plant polyketides results from the number of starter substrates that can be used by the PKSs and from subsequent modifications *via* regio-specific condensation, cyclization, aromatization, hydroxylation, glycosylation, acylation, prenylation, sulfation, and methylation reactions [6]. Compound backbones generated by these PKSs include chalcones, stilbenes, phloroglucinols, resorcinols, benzophenones, biphenyls, bibenzyls, chromones, acridones, pyrones, and curcuminoids [7]. Some of the resulting plant polyketides have been shown to possess anticancer, antimicrobial, antiviral, antioxidant, neuroprotective and oestrogenic activities [8–12]. Such potential health-protecting effects of plant polyketides have stimulated the elucidation of their biosynthetic pathways and the development of frameworks for commercial production.

For industrial or pharmaceutical applications, the use of plant polyketides is mainly limited by their availability [13]. Significant engineering work has been carried out in recent years to increase the yield of polyketides (mainly chalcones and stilbenes) in plants [14, 15]. As is the case for other plant metabolites, many polyketides tend to accumulate in small amounts and may require long growth periods to do so [16]. Purification can also be problematic, as multiple structurally similar metabolites are often present [17]. Total or semi-synthetic approaches are generally challenging and may result in racemic mixtures with relatively low yields [18, 19].

Alternatively, microbes can be utilized as heterologous hosts for polyketides biosynthesis, with several advantages compared to plant and chemical synthesis. Microbes can be grown on inexpensive substrates and have very fast production cycles compared to plants. Current production methods result in microbial synthesis being significantly more environmentally friendly than chemical synthesis. Reconstruction of a plant biosynthetic pathway in microbes is still a challenging task. It first requires the stable introduction of multiple heterologous genes in the microbial host. These genes then have to be expressed and generate functional enzymes. Once functionality of the heterologous pathway has been demonstrated, the main challenge remains in reaching yields sufficient for commercialisation.

This review presents the recent development of microbial engineering for the biosynthesis of plant polyketides, yield improvement and product diversification. Current limitations and bottlenecks are also covered.

## Polyketide biosynthesis in plants

Polyketides are a large group of metabolites found in bacteria, fungi and plants, which are synthesized from acyl-CoA precursors by PKS [20]. PKSs can be grouped in three distinct classes based on their biochemical features and product structure [21]. Type I refers to large modular and multifunctional enzymes, whereas type II PKSs are dissociable complexes usually composed of monofunctional enzymes that are found in bacteria [22]. Plant PKSs are part of the type III group, which comprises homodimeric enzymes of relatively small size [7]. Type III PKSs are also found in bacteria [23] and fungi [24]. Type III PKSs catalyze iterative decarboxylative condensations of malonyl units with a CoA-linked starter molecule [25]. A typical type III PKS reaction involves the loading of a starter molecule, the extension of the polyketide chain and cyclization of the linear intermediate [5]. A great variety of CoA-linked starter substrates can be utilized by plant type III PKSs: acetyl-CoA, malonyl-CoA, methyl-malonyl-CoA, *p*-coumaroyl-CoA, cinnamoyl-CoA, *N*-methylanthraniloyl-CoA, *n*-hexanoyl-CoA, isobutyryl-CoA, isovaleryl-CoA and 3-hydroxybenzoyl-CoA to name a few [5, 7].

**Figure 1:**
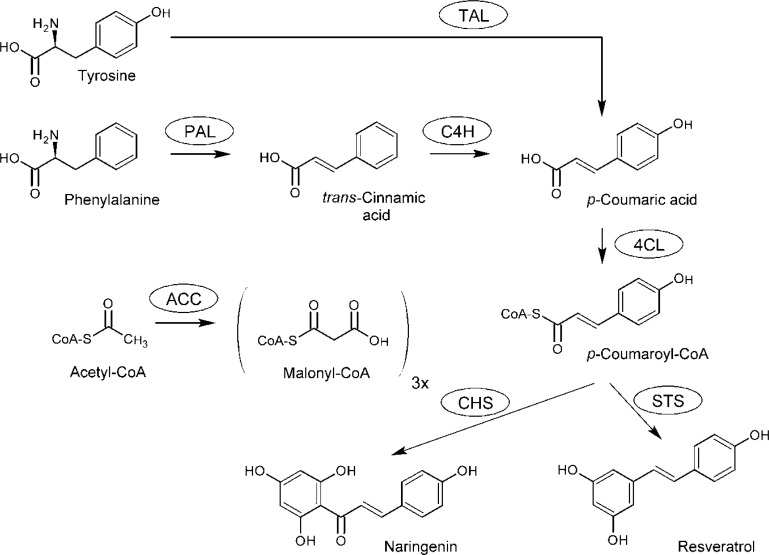
Naringenin chalcone and resveratrol common biosynthetic pathway. TAL, tyrosine ammonia lyase; PAL, phenylalanine ammonia lyase; C4H, cinnamate 4-hydroxylase; 4CL, 4-coumaroyl:CoA ligase; ACC, acetyl-CoA carboxylase; CHS, chalcone synthase; STS, stilbene synthase.

Chalcone synthase (CHS) is the archetypal plant-specific type III PKSs that catalyzes sequential condensation of *p*-coumaroyl-CoA with three molecules of malonyl-CoA to produce naringenin chalcone via a Claisen cyclization reaction (Fig. 1) [26]. Synthesis of *p*-coumaroyl-CoA requires the action of three enzymes. Phenylalanine ammonia lyase (PAL) catalyses the first reaction of the phenylpropanoid pathway by the deamination of phenylalanine into *trans*-cinnamic acid [27]. A cinnamate 4-hydroxylase (C4H) then catalyses the hydroxylation of *trans*-cinnamic acid to yield *p*-coumaric acid [28]. Alternatively, *p-*coumaric acid can be produced directly from tyrosine by a tyrosine ammonia lyase (TAL), bypassing the C4H intermediate [6]. While some PALs have demonstrated promiscuity towards tyrosine, distinct TALs are found in grasses and non-plant organisms [29]. A 4-coumaroyl:CoA ligase (4CL) activates *p*-coumaric acid through the addition of a CoA unit to generate *p-*coumaroyl-CoA. These three enzymes, PAL, C4H and 4CL, are required for normal growth and development and, are therefore highly conserved among plants. A significant fraction of *p*-coumaroyl-CoA is consumed for lignin synthesis, but it is also required for flavonoid production [30]. Phenylpropanoid pathway intermediates are also redirected into synthesis of benzoates, salicylates, coumarins, monolignols, lignans, and phenylpropenes [29]. The other CHS co-substrate, malonyl-CoA, is formed by the carboxylation of acetyl-CoA, a reaction catalyzed in the cytosol by a homomeric acetyl-CoA carboxylase (ACC) [31]. Malonyl-CoA is also required for the synthesis of fatty acids, but is produced in the plastid by a distinct ACC for this purpose [32]. After formation of naringenin chalcone (also called chalconaringenin) by CHS, a variety of flavonoids are created by the combined actions of functionalizing enzymes, namely isomerases, reductases, hydroxylases, glycosyltransferases, acyltransferases, methyltransferases and prenyltransferases [19, 29, 33]. Stilbene synthase (STS), another well studied representative of the plant type III PKSs, uses the same substrates as CHS, but performs an aldol cyclization reaction to produce resveratrol [34] (Fig. 1). STS is found in few diverse plants, such as grapevine, and would have evolved from CHS by a limited number of amino acids substitutions [35].

**Figure 2:**
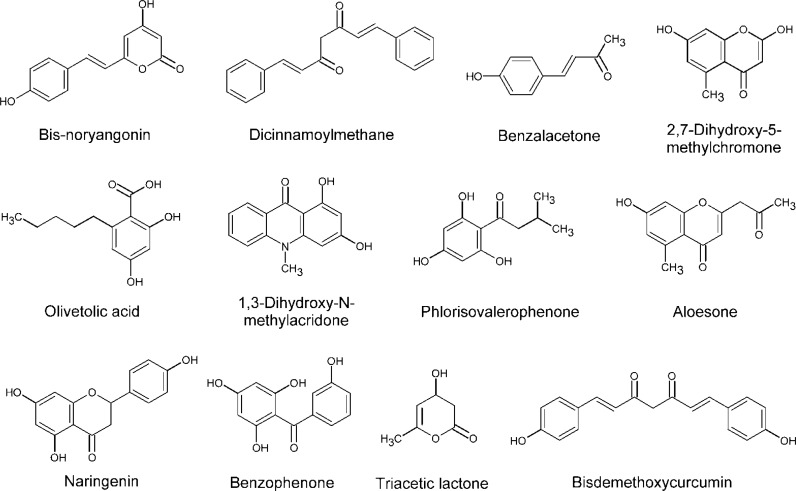
Examples of plant polyketides.

Naringenin chalcone and resveratrol are not the only plant polyketides that can result from the condensation of *p*-coumaroyl-CoA and malonyl-CoA. Different polyketides can be synthesised by varying the number of malonyl-CoA and *p*-coumaroyl-CoA molecules used in the elongation step (Fig. 2). The type of cyclization or its absence also influences the resulting product [7]. Styrylpyrone synthase (SPS) produces bisnoryangonin from the condensation of two molecules of malonyl-CoA [36]. Benzalacetone synthase (BAS) catalyzes the condensation of only one molecule of malonyl-CoA without a cyclization, resulting in diketide benzalacetone [37]. Similarly, curcuminoid synthase (CUS) uses one molecule of malonyl-CoA without cyclization, but produces bisdemethoxycurcumin by the condensation with *p*-coumaroyl-CoA [38]. Other plant PKSs catalyze the condensation of three malonyl-CoA units while employing varying starter substrates. Such PKSs include acridone synthase (ACS), phlorisovalerophenone synthase (VPS), benzophenone synthase (BPS), olivetol synthase (OLS) and 2-pyrone synthase (2PS) [7].

## Engineered microbes

Like many plant secondary metabolites, polyketides are often difficult to extract in large amounts or to synthesize chemically. For those reasons, engineering microbes for the production of plant metabolites is emerging as an advantageous alternative. This approach typically required the construction of new metabolic pathways in the microbial host and the modification of existing ones to enhance productivity. Metabolic engineering for plant polyketide production has been conducted mainly in the two workhorse organisms *Escherichia coli* and *Saccharomyces cerevisiae*. These two microbial hosts are easy to grow and manipulate, are genetically and biochemically well characterized, and have many genetic tools available. As a eukaryote, *S*. *cerevisiae* presents some distinct advantages over *E*. *coli* for the reconstruction of plant pathways. *S*. *cerevisiae* has compartments similar to plant cells and can post-translationally modify proteins. The eukaryotic cellular environment is also more adequate for the expression of functional membrane proteins, such as cytochrome P450s.

*E. coli* and *S. cerevisiae* naturally produce malonyl-CoA, but lack most of the CoA-ester starter substrates needed for plant polyketide synthesis. Although precursors can be supplemented in the growth medium, the production of these substrates represents an important part of the microbe engineering process. Most plant polyketide pathway engineering in microbes described in the literature is based on CHS and STS, with only few other examples using curcuminoid synthase and tetraketide synthase (Table 1).

**Table 1:** Production of plant polyketides in microorganisms.

Host organism	Biosynthetic components	Fed precursor	End-product	Yield (mg/L)	Ref.
*E. coli*	PAL (*Rhodotorula rubra*), 4CL (*Streptomyces coelicolor*), CHS (*Glycyrrhiza echinata*)	Tyrosine	Naringenin	0.453	[39]
		Phenylalanine	Pinocembrin	0.752	
	TAL (*Rhodobacter sphaeroides*), 4CL, CHS (*Arabidopsis thaliana*)	None	Naringenin	20.8	[40]
	PAL (*R. rubra*), 4CL (*S. coelicolor*), CHS (*G. echinata*), CHI (*Pueraria lobata*), ACC (*Corynebacterium glutamicum*)	Tyrosine	Naringenin	57	[41]
		Phenylalanine	Pinocembrin	58	
	4CL (*A. thaliana*), STS (*Arachis hypogaea*)	*p*-Coumaric acid	Resveratrol	104.5	[42]
	4CL (*Nicotiana tabacum*), STS (*Vitis vinifera*)	*p*-Coumaric acid	Resveratrol	16	[43]
	4CL, FSI (*Petroselinum crispum*), CHS, CHI (*Petunia* × *hybrida*), OMT (*Mentha* × *piperita*)	*p*-Coumaric acid	Apigenin	0.415	[44]
		Caffeic acid	Luteolin	0.01	
		*p*-Coumaric acid	Genkwanin	0.208	
	4CL (*P. crispum*), CHS (*P*. × *hybrida*), CHI (*Medicago sativa*), ACC (*Photorhabdus luminescens*), ACS (*E. coli*)	Cinnamic acid	Pinocembrin	429	[45]
		*p*-Coumaric acid	Naringenin	119	
		Caffeic acid	Eriodictyol	52	
	4CL (*Lithospermum erythrohizon*), CHS, CHI (*G. echinata*), STS (*A. hypogaea*), FNS (*P*. *crispum*), F3H, FLS (*Citrus*), ACC (*C. glutamicum*)	*p*-Coumaric acid	Flavones	84	[46]
			Flavonols	33	
			Stilbenes	171	
		Fluorocinnamic acid	Flavanones	102	
	4CL (*P. crispum*), CHS, CHI (*P*. × *hybrida*), MatB, MatC (*Rhizobium trifolii*)	Cinnamic acid	Pinocembrin	480	[47]
		*p*-Coumaric acid	Naringenin	155	
		Caffeic acid	Eriodictyol	50	
	PAL (*R. rubra*), 4CL (*L. erythrorhizon*), CUS (*Oryza sativa*)	Tyrosine	Bisdemethox-ycurcumin	53.4	[48]
		Phenylalanine	Dicinnamoyl-methane	107	
		Phenylalanine/ tyrosine	Cinnamoyl-p-coumaroylme-thane	19.2	
		Ferulic acid	Curcumin	113	
	TAL (*Rhodotorula glutinis*), 4CL (*P. crispum*), CHS (*P*. × *hybrida*), CHI (*M. sativa*)	None	Naringenin	84	[13]
	TAL (*Saccharothrix espanaensi*), 4CL (*S*. *coelicolor*), CHS (*A. thaliana*), STS (*A. hypogaea*)	None	Naringenin	5.3	[49]
			Resveratrol	1.4	
	4CL (*P. crispum*), CHS (*P*.× *hybrida*), CHI (*M. sativa*), ACC (*P. luminescens*), PGK, PDH (*E. coli*)	*p*-Coumaric acid	Naringenin	474	[50]
	4CL (*P. crispum*), CHS (*P*. × *hybrida*), CHI (*M. sativa*), F3H (*A. thaliana*), OMT (*Streptomyces avermitilis*), ACC, ACS (*Nocardia farcinica*)	*p*-Coumaric acid	7-O-Methyl aromadendrin	2.7	[51]
	TAL (*R. glutinis)*, 4CL (*P. crispum*), CHS (*P*. × *hybrida*), CHI (*M. sativa*), AroF, PheA (*E*. *coli*), MatB, MatC (*R. trifolii*)	None	Pinocembrin	40	[52]
*S. cerevisiae*	4CL (*Populus trichocarpa* × *Populus deltoides*), STS (*V. vinifera*)	*p*-Coumaric acid	Resveratrol	1.45x10^-3^	[53]
	PAL (*Rhodosporidium toruloides*), 4CL (*A. thaliana*), CHS (*Hypericum androsaemum*)	None	Naringenin	7	[54]
			Pinocembrin	0.8	
	C4H (*A. thaliana*), 4CL (*P. crispum*), CHS, CHI (*P*. × *hybrida*)	Cinnamic acid	Pinocembrin	16.3	[55]
		*p*-Coumaric acid	Naringenin	28.3	
		Caffeic acid	Eriodictyol	6.5	
	C4H (*A. thaliana*), 4CL, FSI (*P. crispum*), CHS, CHI (*P*. × *hybrida*),	Cinnamic acid	Chrysin	-	[56]
		*p*-Coumaric acid	Apigenin	-	
		Caffeic acid	Luteolin	-	
	4CL (*N. tabacum*), STS (*V. vinifera*)	*p*-Coumaric acid	Resveratrol	6	[43]
	Fusion of 4CL (*A. thaliana*) and STS (*V. vinifera*)	*p*-Coumaric acid	Resveratrol	5.25	[57]
	PAL, CPR (*P. trichocarpa* × *P. deltoides*), C4H, 4CL (*Glycine max*), STS (*V. vinifera*)	Phenylalanine	Resveratrol	0.31	[58]
	4CL (*A. thaliana*), STS (*V. vinifera*)	*p*-Coumaric acid	Resveratrol	391	[59]
	4CL (*A. thaliana*), STS (*A. hypogaea*)	*p*-Coumaric acid	Resveratrol	3.1	[60]
	TAL (*R. sphaeroides*), fusion of 4CL (*A. thaliana*) and STS (*V. vinifera*)	Tyrosine	Resveratrol	1.9	[61]
	OAC, TKS (*Cannabis sativa*)	Hexanoic acid	Olivetolic acid	0.48	[62]
	PAL (*R*. *toruloides*), C4H, 4CL (*A*. *thaliana*), STS (*A. hypogaea*), ACC (*S*. *cerevisiae*)	None	Resveratrol	4.3	[63]
		Tyrosine		5.8	
	PAL, C4H, CPR, 4CL, CHS, CHI (*A*. *thaliana*), TAL (*Rhodobacter capsulatus*), ARO4^G226S^ (*S. cerevisiae*)	None	Naringenin	109	[64]
*Streptomyces venezuelae*	4CL (*S. coelicolor*), CHS (*A*. *thaliana*), STS (*A. hypogaea*)	Cinnamic acid	Pinocembrin	6	[65]
		*p*-Coumaric acid	Naringenin	4	
			Resveratrol	0.4	

## Chalcone synthases

Hwang *et al*. [39] were the first to report the production of plant polyketides in *E*. *coli*. Naringenin and pinocembrin were produced by co-expressing PAL from the yeast *Rhodotorula rubra*, 4CL from the actinobacterium *Streptomyces coelicolor* A3(2), and CHS from the plant *Glycyrrhiza echinata*. PAL could use both tyrosine and phenylalanine as substrates, yielding *p*-coumaric acid and *trans*-cinnamic acid, respectively. Similarly, 4CL catalyzed the activation of *trans*-cinnamic and *p*-coumaric acid, and CHS used both cinnamoyl- CoA and *p*-coumaroyl-CoA as starters. Very small amounts of naringenin (0.27 µg/L) and pinocembrin (0.17 µg/L) were detected in the culture medium. To increase yields, tyrosine and phenylalanine were supplemented. As a result, yields of naringenin and pinocembrin were increased to 452.6 µg/L and 751.9 µg/L, respectively. The same group was then able to improve production of naringenin and pinocembrin by overexpressing an ACC from *Corynebacterium glutamicum* and by optimising culture conditions [41]. They also added a chalcone isomerase from *Pueraria lobata* to catalyze the isomerisation of naringenin chalcone to naringenin. Although the malonyl-CoA concentrations were not reported, these results suggested that an increase in the amount of malonyl-CoA, as a result of ACC overexpression, led to improved production of polyketides. Under culture conditions where *E. coli* cells were concentrated to 50 g/L, yields of naringenin and pinocembrin reached about 60 mg/L in the presence of tyrosine and phenylalanine, respectively. Watts *et al*. [40] were the first to use a true TAL enzyme from the bacteria *Rhodobacter sphaeroides*. This enzyme, while accepting both tyrosine and phenylalanine as substrates, exhibits a much higher specificity toward tyrosine [66]. By using TAL, *p*-coumaric acid was efficiently produced directly from tyrosine without producing *trans*-cinnamic acid and pinocembrin.

Jiang *et al*. [54] first described the production of naringenin and pinocembrin in *S*. *cerevisiae*. This was accomplished by the combined expression of PAL from the red yeast *Rhodosporidium toruloides*, 4CL from *Arabidopsis thaliana*, and CHS from the plant *Hypericum androsaemum*. As described by Miyahisa *et al*. [41], PAL showed tyrosine ammonia lyase activity, which enabled the biosynthesis of naringenin without the need of C4H. Precursor feeding studies indicated that the tyrosine pool size limited *p*-coumaric acid production, although no additional naringenin could be produced. Yan *et al*. [55] opted for a different approach, using four plant-derived genes: C4H from *Arabidopsis thaliana*, 4CL from *Petroselinum crispum*, CHS and chalcone isomerase (CHI) from *Petunia* × *hybrida*. Feeding assays were done with *trans*-cinnamic acid, *p*-coumaric acid, caffeic acid, and ferulic acid, yielding respectively pinocembrin (16.3 mg/L), naringenin (28.3 mg/L), eriodictyol (6.5 mg/L), but no homoeriodictyol. However, naringenin yield was quite low (0.2 mg/L) when *trans*-cinnamic acid was the precursor, indicating that C4H was a rate-limiting step. In a follow-up study, the same group tested two flavone synthases (FSI and FSII) in their yeast strain harbouring C4H, 4CL, CHS, and CHI [56]. The resulting strains converted fed phenylpropanoid acid precursors into the flavone molecules chrysin, apigenin, and luteolin. Yields were increased by overexpressing the yeast cytochrome P450 reductase (CPR1) and by using acetate as carbon source. CPR1 overexpression probably improved both C4H and FSII activities. The overall increase in flavone production with acetate was hypothesized to result from increased carbon flux toward malonyl-CoA. Leonard *et al*. [44] also evaluated flavone production in *E. coli*, using 4CL and FSI from *Petroselinum crispum*, CHS and CHI from *Petunia* x *hybrida*, and 7-*O*-methyltransferase (OMT) from *Mentha* x *piperita*. *E*. *coli* successfully produced apigenin, luteolin and genkwanin from fed phenylpropanoid acid precursors.

Results from Miyahisa *et al*. [41] and Leonard *et al*. [56] indicated that malonyl-CoA was most probably limiting polyketide production in *E. coli* and *S. cerevisiae*. Leonard *et al*. [45] explored various strategies to increase the intracellular pool of malonyl-CoA in *E. coli*. Overexpression of four ACC subunits from the bacteria *Photorhabdus luminescens* resulted in an increase in flavone production, especially for pinocembrin. Due to ACC being a biotin-dependent enzyme, yields were further increased by combining expression of ACC with a biotin ligase (BL). Three BLs were tested, one from *P*. *luminescens*, one from *E. coli*, and a fusion of those two. The co-expression of ACC and BL from *P*. *luminescens* gave the best result. In another approach, flux toward malonyl-CoA was increased through the amplification of acetate assimilation. Culture medium was supplemented with acetate in addition to glucose, and acetyl-CoA synthetase (ACS) from *E. coli* was overexpressed along with ACC. ACS catalyses the formation of acetyl-CoA from acetate and CoA. With this last approach, pinocembrin, naringenin, and eriodictyol accumulated in the culture medium to 429 mg/L, 119 mg/L, and 52 mg/L, respectively.

Leonard *et al*. [47] used a more elaborate metabolic engineering approach for producing flavanones from exogenous phenylpropanoid acids in *E. coli*. To increase availability of malonyl-CoA, a malonyl-CoA synthetase (MatB) and a putative malonate transporter (MatC) from the bacteria *Rhizobium trifolii* were introduced in *E. coli*. MatB and MatC allowed the production of malonyl-CoA from exogenous malonate, bypassing the natural metabolism from glucose. The introduction of this malonate assimilation pathway increased production of naringenin, pinocembrin and eriodictoyl by 269%, 1555% and 355%, respectively. Yields were further increased by attenuating the activity of fatty acid metabolism, which competes for malonyl-CoA. This was done by adding a fatty acid synthase inhibitor to the culture medium.

Park *et al*. [65] reported the first plant-derived polyketide production in an organism other than *E. coli* and *S. cerevisiae*. Genes encoding 4CL from *Streptomyces coelicolor* and CHS from *A*. *thaliana* were expressed in *Streptomyces venezuelae*. Relatively small amounts of naringenin (4 mg/L) and pinocembrin (6 mg/L) were produced when fed with *p*-coumaric acid and *trans*-cinnamic, respectively.

In most of the strategies described previously, polyketide production required supplementation of precursors. Santos *et al*. [13] engineered a four-step pathway consisting of a TAL, 4CL, CHS, CHI in a tyrosine-overproducing *E. coli* strain, allowing naringenin production directly from glucose. Enzymes from different sources were tested, and genes were codon-optimized for *E*. *coli* expression. The performance of TAL from *R. sphaeroides* (RsTAL) and from the yeast *Rhodotorula glutinis* (RgTAL) was evaluated in the absence of the other downstream enzymes. Only small amounts of *p-*coumaric acid (1.5-5.5 mg/L) were produced with RsTAL compared to RgTAL (104-213 mg/L). Enzymatic assays with purified enzymes revealed a twelve-fold higher catalytic activity with tyrosine for RgTAL [66]. Different 4CLs, CHSs and CHIs were also evaluated. The most efficient strain was obtained with RgTAL, 4CL from *Petroselinum crispum*, CHS from *Petunia hybrida* and CHI from *Medicago sativa* L. Without any precursor supplementation, the strain was capable of producing 29 mg/L of naringenin and up to 84 mg/L with the addition of cerulenin, a fatty acid enzyme inhibitor. Choi *et al*. [49] also reported the production of naringenin in *E*. *coli* without feeding tyrosine or *p*-coumaric acid. Their synthetic gene cluster was composed of TAL from *Saccharothrix espanaensi*, 4CL from *S. coelicolor* and CHS from *A. thaliana*. The yield of naringenin obtained (5.3 mg/L) was low compared to Santos *et al*. [13], as no further metabolic engineering was done to improve precursors level.

With the aim to improve the availability of malonyl-CoA in *E. coli*, Xu *et al*. [50] used an integrated computational and experimental approach. A customized version of the OptForce algorithm was utilized to predict a minimal set of metabolic interventions [67]. Based on that model, genes encoding a fumarase (*fumC*) and a succinyl-CoA synthetase (*sucC*) were knocked out. FumC and SucC are part of the TCA cycle and catalyze the interconversion of fumarate to malate and the reversible reaction of succinyl-CoA to succinate, respectively. Knocking out those genes partially inactivated the TCA cycle, which in turn increased naringenin production by 30% compared to the wild type strain. The model also identified target genes that if upregulated would augment the production of the precursor acetyl-CoA. By co-expressing acetyl-CoA carboxylase (ACC) with either phosphoglycerate kinase (PGK) or glyceraldehyde-3-phosphate dehydrogenase (GAPD), and pyruvate dehydrogenase (PDH), production of naringenin was increased by about 220%. Finally, overexpression of ACC, PGK and PDH in the double knockout strain resulted in the production of 474 mg/L of naringenin, the highest yield achieved so far.

Koopman *et al*. [64] were the first to engineer *S. cerevisiae* for improved tyrosine availability for the production of naringenin. Synthesis of tyrosine was increased by introducing a feedback resistant 3-deoxy-d-arabinose-heptulosonate-7-phosphate (DAHP) synthase allele (*ARO4* ^*G226S*^) and deleting the other allele (*ARO3*). Additionally, the loss of tyrosine to side products was reduced by deleting the phenylpyruvate decarboxylases (Aro10, Pdc5, Pdc6). Most of the naringenin biosynthetic pathway parts (PAL, C4H, CPR, 4CL, CHS, CHI) were from *A. thanliana*, but a TAL from *R. capsulatus* was also used. The presence of PAL, C4H, CPR and TAL allowed synthesis of *p*-coumaric acid from both tyrosine and phenylalanine. In shake-flask cultures, approximately 50 mg/L of naringenin was produced directly from glucose and, more than 100 mg/L in controlled aerobic batch cultures.

Wu *et al*. [52] engineered *E. coli* to increase both the supply of phenylalanine and malonyl-CoA. Carbon flux toward phenylalanine was increased by overexpressing 3-deoxy-d-arabinose-heptulosonate-7-phosphate (AroF) and a feedback-inhibition-resistant chorismate mutase/prephenate dehydratase (PheA). The supply of malonyl-CoA was increased by introducing the malonate assimilation pathway from *R. trifolii* (MatB and MatC), as described previously by Leonard *et al*. [47]. The resulting strain, in which TAL (*R. glutinis)*, 4CL (*P. crispum*), CHS (*P*. × *hybrida*) and CHI (*M. sativa*) were co-expressed, allowed the production of pinocembrin (40 mg/L) without any precursor supplementation.

## Stilbene synthases

Becker *et al*. [53] were the first to report the production of resveratrol in an engineered microorganism. The resveratrol (1.45 µg/L) was produced in *S*. *cerevisiae* from fed *p*-coumaric acid by co-expressing 4CL from *Populus trichocarpa* × *Populus deltoides* and STS from *Vitis vinifera*. Zhang *et al*. [57] further improved resveratrol yield in *S. cerevisiae* by fusing 4CL from *A. thaliana* and STS from *V. vinifera*. The 4CL::STS fusion protein increased resveratrol production by up to 15-fold compared to the co-expression of 4CL and STS. Nevertheless, the yield of resveratrol (5.25 mg/L) obtained from *p*-coumaric acid feeding remained relatively low. In *E. coli*, resveratrol was produced at a higher level. Concentrations reached 16 mg/L by co-expressing 4CL from *Nicotiana tabacum* and STS from *V. vinifera* [43], and over 100 mg/L when co-expressing 4CL from *A. thaliana* and STS from *Arachis hypogaea* [42]. A similar two-reaction approach was used in *S. venezuela* with a 4CL from *S. coelicolor* and a STS from *A. hypogaea*, producing 0.4 mg/L of resveratrol [65]. Trantas *et al*. [58] extended the synthetic pathway in *S. cerevisiae* by adding a PAL and a CPR from *Populus trichocarpa* x *P. deltoides* and a C4H from *Glycine max*. This extended pathway synthesized resveratrol from fed phenylalanine, but with a yield of less than 1 mg/L. Sydor *et al*. [59] evaluated resveratrol synthesis from *p-*coumaric acid in different *S. cerevisiae* strains expressing 4CL from *A. thaliana* and STS from *V. vinifera*. Different growth media were also tested. In synthetic medium (SD), the strain CEN.PK2-1 produced 6 mg/L of resveratrol. When grown in the rich medium YEPD, yield increased dramatically to 262 mg/L. They also evaluated four industrial yeast strains for their efficiency in resveratrol production. Three strains produced resveratrol in various concentrations, but one metabolized *p*-coumaric acid and did not synthesis any resveratrol. The highest yield (391 mg/L) was obtained with an *S. cerevisiae* strain isolated from a Brazilian sugar cane plantation. Other attempts to produce resveratrol in *E. coli* and *S. cerevisiae* have resulted in yields not exceeding 6 mg/L [49, 60, 61, 63].

## Curcuminoid synthase

Curcuminoid synthase (CUS) is a type III PKS involved in the formation of diarylheptanoid compounds, such as those found in the spice turmeric [68]. An artificial curcuminoid biosynthetic pathway based on a CUS from rice (*Oryza sativa*) was assembled in *E. coli* [48]. CUS catalyzes the formation of bisdemethoxycurcumin from two molecules of *p*-coumaroyl-CoA and one molecule of malonyl-CoA [68]. It also accepts cinnamoyl-CoA and feruloyl-CoA as substrates to produce dicinnamoylmethane and curcumin, respectively. Along with CUS, a PAL from *R. rubra* and a 4CL from *Lithospermum erythrorhizon* were assembled in *E. coli*. The resulting strain produced bisdemethoxycurcumin (53.4 mg/L), dicinnamoylmethane (107 mg/L) and cinnamoyl-*p*-coumaroylmethane (19.2 mg/L) from supplemented tyrosine and/or phenylalanine. Curcumin (113 mg/L) was also synthesised when ferulic acid was fed.

## Tetraketide synthase and olivetolic acid cyclase

Gagne *et al* [62] have recently reported the identification of a novel polyketide cyclase enzyme, olivetolic acid cyclase (OAC), which functions together with a type III PKS to form olivetolic acid using hexanoyl-CoA as a starter substrate. Olivetolic acid is the first polyketide intermediate in the cannabinoid biosynthetic pathway in *Cannabis sativa* (marijuana, hemp). Co-expression of a type III PKS from cannabis, dubbed tetraketide synthase (TKS) based on its presumed synthesis of a linear tetraketide, and OAC in yeast that was supplied with exogenous hexanoic acid allowed for the production of small amounts of olivetolic acid (0.48 mg/L). Further optimization of precursor supply, including the use of hexanoyl-CoA synthetases [69] to augment the supply of the starter substrate, may allow increased yield of this alkylresorcinolic product.

## Limitations and bottlenecks

As with other secondary metabolites, achieving high yields of plant polyketides in microbes is a challenging task. An array of factors can impair productivity. The most obvious include the functionality of the biosynthetic pathway and the availability of precursors. The efficacy of a heterologous pathway is first affected by the concentration of its constituent enzymes. Although transcription rate can impact gene expression, there is no general correlation between mRNA and protein abundance [70–72]. Enzyme concentration is also influenced by translation-related mechanisms and protein degradation rate [73]. Codon usage is known to affect heterologous gene expression [74, 75]. Although codon optimization does not necessarily improve expression, it should be part of every metabolic engineering approach. In addition to expression, enzyme efficiency (k_cat_/K_M_) can affect final productivity. Enzyme stability in the heterologous host, cofactor requirements, post-translational modification and regulation, subcellular compartmentation and feedback inhibition are other factors to consider. If possible, multiple enzymes should be tested to find the best candidates for each step of the pathway. In plants, polyketide pathways are tightly regulated so that only the required quantities of metabolites are produced [76]. In contrast, synthetic pathways assembled in heterologous hosts are not under such regulatory control. This absence of regulation can lead to growth retardation and may cause accumulation of toxic intermediates. Modulating expression of relevant genes is a commonly used strategy for balancing pathway dynamics. This can be done by tuning promoter strengths, by manipulating non-coding regions, and by altering gene or plasmid copy number [77–79]. Rate-limiting enzymes can be substituted for more efficient ones or can be improved by protein engineering [80, 81].

The productivity of a synthetic pathway is greatly determined by the supply of precursor metabolites. As demonstrated by multiple research groups, tyrosine/phenylalanine and malonyl-CoA are limiting for the production of polyketides in microbes [39, 41, 45, 47, 50, 54]. Although tyrosine and phenylalanine can be supplemented in the growth medium, this is not a sustainable option for cost-effective polyketide production. A more viable alternative is to increase the pool of tyrosine/phenylalanine within the polyketide-producing host, an approach successfully used by Santos *et al*. [13]. Different groups succeeded in increasing tyrosine production in *E. coli* and *S. cerevisiae* by overproducing feedback-inhibition-resistant enzymes from the aromatic amino acid biosynthesis pathway and by removing transcriptional regulation [82–85]. Santos *et al*. [86] subsequently improved the tyrosine titer in *E. coli* by using a global transcription machinery engineering approach (gTME) [87]. A common approach to increase the pool of malonyl-CoA is to overexpress ACC, the enzyme that catalyzed its synthesis from acetyl-CoA [41, 45, 63, 88]. Malla *et al*. [51] also included an acetyl-CoA synthetase to increase the carbon flux toward malonyl-CoA. Alternatively, malonyl-CoA can be synthesised from malonate by the co-expression of a malonyl-CoA synthetase (MatB) and a putative dicarboxylate carrier protein (MatC) from *Rhizobium trifolii*, a strategy succesfully used in *E. coli* [47]. More sophisticated engineering approaches, that included the overexpression and the knockout of multiple genes, were also used to increase the pool of malonyl-CoA [50, 89, 90].

## Combinatorial biosynthesis of novel, unnatural plant type III polyketides

Type III polyketides are of particular interest due to their potential as bioactive compounds with therapeutic uses. When screening for desired functionalities, the larger the library of diverse compounds one can create, the higher the chances of finding a compound that can fulfill a purpose of interest. In addition to the role of the type III PKSs, the diversity of plant polyketides also arises from the range of tailoring enzymes that perform post-polyketide modifications [91]. Because of this, the use of combinatorial biosynthesis is an ideal way to increase variety in an ever expanding library of polyketides derived from type III PKSs. Combinatorial biosynthesis entails designing artificial biosynthetic pathways containing enzyme-encoding genes from different species and/or organisms into a heterologous host. In this way, PKSs can be combined with tailoring enzymes from other organisms resulting in polyketides that would otherwise never be formed.

In 1999 McDaniel *et al*. [92] were able to create a library of over 50 unnatural products (macrolides) by genetically modifying the erythromycin PKS genes thereby combinatorially affecting their catalytic activities in the biosynthetic pathway. Five years later, Katsuyama *et al*. [46] used combinatorial biosynthesis and precursor-directed biosynthesis to create novel plant polyketides. This required a multi-plasmid approach, transforming *E. coli* with three plasmids each containing three distinct sections of the polyketide biosynthetic pathway. The first plasmid (substrate synthesis plasmid) contained 4CL from *Lithospermum erythrohizon*. The second plasmid (polyketide synthesis plasmid) contained CHS from *Glycyrrhiza echinata* and CHI genes from *Pueraria lobata*, or contained the STS gene from *Arachis hypogaea*. The post-polyketide modification plasmid contained either genes for flavone (FNS I gene from *Petroselinum crispum*) or flavonol production (flavanone 3β-hydroxylase (F3H) and flavonol synthase (FLS) genes from *Citrus*). The various genes from various organisms used to create this pathway define the combinatorial biosynthesis aspect of this experiment. Precursor-directed biosynthesis is a technique where cells are supplied with unnatural precursors which result in the formation of unnatural products. Natural and unnatural carboxylic acids were also exogenously introduced to the recombinant *E. coli*. When natural carboxylic acids were introduced, the system created corresponding natural polyketides. When unnatural carboxylic acids were introduced, the organism mainly produced the corresponding unnatural polyketides. The authors hypothesized that the reason for this was the relatively relaxed substrate specificity of their FNS I. This system resulted in the synthesis of 14 flavanones, 13 flavones, 8 flavanols, and 15 stilbenes at high yields and, the production of 20 triketide pyrones and 17 tetraketide pyrones through derailment or incorrect cyclization. Out of the 50 polyketides, 36 were novel compounds.

Also in 2007, Chemler *et al*. [93] were able to create a type III polyketide pathway in *Saccharomyces cerevisiae* capable of utilizing acrylic acid analogues to produce novel unnatural polyketides. In this study, acrylic acid analogues were first screened *in vitro* for their potential as substrates for the flavonoid enzymes. In the second-stage screening, the compounds were tested for their ability to be synthesized into flavanone analogues through an *in vitro* assay where 4CL, CHS and CHI from petunia and a malonyl-CoA synthetase from *Rhizobium trifolii* were provided. The acrylic acids that passed both screenings were then provided as substrates for a *S. cerevisiae* strain expressing 4CL, CHS and CHI on a plasmid. This system resulted in the expression of six unnatural flavanones. In an effort to increase the amount of novel flavonoids produced, a plasmid containing flavanone 3β-hydroxylase (FHT) from *Malus domestica* was added to the initial strain, creating six novel dihydroflavonols from 6 acrylic acids.

Another interesting approach to the formation of novel polyketide compounds is the manipulation or mutation of the type III PKS protein itself. In 2007, Abe *et al*. [94] mutated the pentaketide chromone synthase (PCS) from *Aloe arborescens* in order to create novel polyketides. *E. coli* expressing a triple mutant (F80A/Y82A/M207G) version of PCS, using malonyl-CoA as a substrate, was found to lead to the production of an unnatural novel nonaketide (naphthopyrone). In addition, control over polyketide chain length from triketide to octaketide has been demonstrated through site-directed mutagenesis of amino acid residues lining the active site of certain PKSs [94–96]. In one case, Abe *et al*. [96] were able to perform site-directed mutagenesis on an octaketide producing PKS, thereby creating the mutant CHS's G207A, G207T, G207M, G207L, G207F, and G207W. The mutagenesis of this single residue led to the formation of smaller polyketides (triketides to heptaketides). Site-directed mutagenesis of a CHS has also been shown to affect its starter molecule specificity. In 2002 Jez *et al*. [97] were able to create a type III PKS capable of using the previously unusable substrate N-methylanthraniloyl-CoA to produce a novel alkaloid. This was accomplished by a single point mutation, F215S, in the PKS.

## Concluding remarks and outlook

With the increasing availability of plant genomic sequences, the repertoire of plant biosynthetic genes is continuously expanding. These biological parts are the starting point for the reconstruction of plant secondary metabolic pathways in microorganisms [3]. The success of a defined engineering approach greatly depends on the use of adequate synthetic enzymes. After establishing the pathway in the microbial host and demonstrating the production of the desired metabolite, the next step is to increase product yield to the point of economic viability. Yield improvement currently represents the greatest engineering challenge. Metabolic limitations and bottlenecks have to be identified, and should be considered from the perspective of the whole organism. A holistic approach including rational and computational analysis is fast becoming the norm, and is advisable before carrying out significant manipulation of metabolic pathways. Not only can such an approach inform planning design, it can also be used iteratively once physiological and yield data is available from initial metabolic systems.

While informed engineering decisions are comparatively easy to make in the canonical model organisms, many promising host species currently lack sufficient characterisation to exploit fully [19, 65, 98]. Along with improved tools for genetic manipulation, as metabolic and regulatory data becomes available for novel hosts, more engineering will take place in these systems to take advantage of differences in their evolutionarily engineered metabolome. Such an expansion of host chassis, both highly engineered and naturally diverse, will undoubtedly complement the current molecular tuning of engineered plant polyketide pathways.

Microbial production of plant polyketides is still at an early stage and is greatly limited by the availability of characterised biosynthetic genes and the lack of knowledge about pathway structures. Recent engineering efforts have focused on CHS and STS, with most of the PKS diversity barely touched. Given all the avenues for development available to researchers, there is significant room for the field to grow.
